# Host-parasite interactions: an interview with Emily Troemel

**DOI:** 10.1186/s12915-018-0592-6

**Published:** 2018-11-01

**Authors:** Emily Troemel

**Affiliations:** Division of Biological Sciences, University of California, San Diego, La Jolla, CA USA

**Keywords:** Infection, Immunity, Host–parasite interactions, *C. elegans*, Pathogens

## Abstract

Emily Troemel is a Professor at the University of California San Diego, where her lab uses *Caenorhabditis elegans* to study host–pathogen interactions and the shaping of the immune response. In this interview, Emily shared her thoughts on peer review and its role in training future scientists, and the possibility of a new form of immunity in epithelia.

## What are your current research interests?

My lab is interested in how epithelial cells respond to intracellular infections caused by co-evolved pathogens. In particular we focus on how the nematode *Caenorhabditis elegans* responds to infection by the microsporidian species *Nematocida parisii*, which is the most common cause of disease for *C. elegans* in the wild [1, 2]. These studies have led to us to investigate commonalities in the response to a natural viral infection as well, and to explore how the response to intracellular infection can improve tolerance of proteotoxic stressors [3, 4].

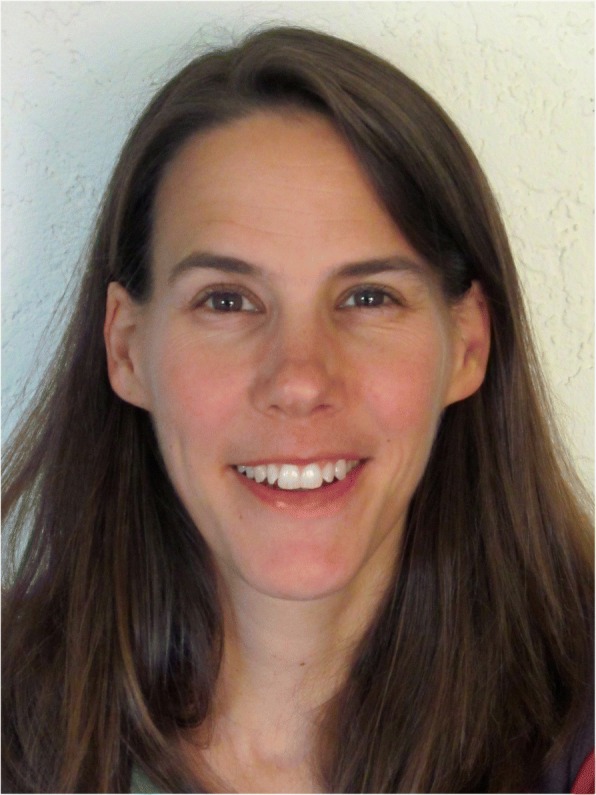



## What are your predictions for the field over the next 5 years?

It may not happen in the next 5 years, but I believe over the next 10–20 years we will find a new type of immunity acting in epithelial cells at the earliest stages of infection. This hypothetical immune response would detect and destroy virulence factors delivered inside host cells using host immune receptors that are species-specific, because pressure from pathogen factors causes these receptors to diversify. Thus, it would be difficult to find conservation on the sequence level for these receptors across phylogeny, but there could be conservation on the functional level. At least I like to dream that we’ll find this novel form of immunity!

## What motivates you to provide peer review for journals?

While our current system of peer review isn’t perfect, I believe that it is an important method for improving the quality of scientific inquiry. The science in my lab has benefited from peer review on many occasions, and I want to pay forward the benefits I have received. I know that many of us take our jobs as peer reviewers very seriously and give it considerable time and effort. In addition to providing a critical assessment of research and ensuring that it meets certain criteria before being published, reviewing papers also provides an opportunity to train new scientists in the lab about how to critically read scientific papers. I often review papers together with a postdoc or grad student in the lab, which I believe improves the quality of the review and gives them an opportunity to learn about the process.

## Have you had any memorably good or bad experiences of peer review, as an author or as a reviewer?

Peer review can be painful, but it is also a privilege and can be beneficial to have the full attention of an expert on your work! One positive experience I had as an author was from reviews we received on the first R01 (NIH Research Project Grant) submission from the lab. Reviewers gave us incredibly detailed critiques and encouragement to pursue the question of how natural microsporidian pathogens exit host cells, as well as how *C. elegans* fights off these natural infections. Looking back at those reviews 9 years later, I appreciate how their feedback was spot on—both for the areas where they had concern and the areas where they encouraged us. I am very grateful to them!

**Website:** http://labs.biology.ucsd.edu/troemel/Lab_website/Home_page.html
